# The acute economic recession: WHO diabetes target 2030 becoming unrealistic in Sri Lanka

**DOI:** 10.1002/hsr2.1027

**Published:** 2022-12-28

**Authors:** Gajarishiyan Rasalingam

**Affiliations:** ^1^ Usher Institute, College of Medicine and Veterinary Medicine University of Edinburgh Edinburgh UK; ^2^ District General Hospital Trincomalee Sri Lanka

**Keywords:** endocrinology and metabolic disorders, health economics and evaluation, health policy, public health, diabetes

In the 75th session of the World Health Assembly in Geneva in May 2022, the WHO member states have amended a new commitment to global diabetes coverage called “Diabetes targets: accelerating progress towards 2030”.^1^ It comprises five targets that include: 80% of people with diabetes are diagnosed, 80% of people have reasonable glycemic control, 80% of diagnosed people have good blood pressure control, 60% of people with diabetes over 40 years receive statins, and 100% of people with type 1 diabetes have access to affordable insulin and blood glucose self‐monitoring. The world leaders pledge to reach these targets over the next 8 years.^1^ The current acute economic crisis in Sri Lanka has burdened the country's health care systems.^2^ This situation widens the gap in reaching this global commitment.

In Sri Lanka, the health system is free, with universal access at its core. More than 95% of Sri Lankan people rely on free public health care services.^3^ The health system has performed consistently better than that of neighboring countries. The COVID‐19 pandemic and the consequences of years of financial mismanagement and political instability now had resulted in the complete economic collapse the country has ever seen.^4^ This subsequently resulted in the depletion of foreign reserve money. The country can no longer afford to pay for imported items such as medicines, 85% of which are imported.^5^ The economic crisis has had a direct influence on patient care. In April 2022, the Government Medical Association declared a national health emergency, stating that the health system would collapse unless there were immediate financial support from foreign countries.^6^

Patients with chronic diseases and multimorbidity have been particularly affected by the economic crises. Medicines for chronic illnesses become a colossal shortage in hospitals.^3^ Moreover, the cost of living has dramatically increased over the last few months, causing extra challenges. This is not a short‐term problem; the economists predict the recovery may take over the next few years.^7^

The scale of the problem can be exemplified by considering the impact on diabetic patients. In Sri Lanka, it is estimated that 1.1 million people live with diabetes and the cost per head is approximately 144.6 USD.^3^ In the current market, a glucose meter costs about 5000 Rs (14 USD) (An increase of 19%), 25 glucose testing strips cost around 2000 Rs (6 USD) (An increase of 11%), and one 10 ml vial of soluble insulin increased from 600 Rs (1.6 USD) to 1930 Rs (5.9 USD) (An increase of 53%).^8^ These prices are very high for the general population. The Government hospitals are running out of medicines, expecting the patient to buy from the private sector. The economic burden already severely impacts individual families. A large proportion of the population will likely not have a choice but to prioritize something else, such as fuel and food, which prices are also steadily increasing.

To overcome this medical crisis, we need a solid and stable government. Extensive economic transformation is necessary to increase the economic growth of the country. As an immediate measure, the government should negotiate with foreign countries and international organizations to import essential medicine. Relief measures such as insulin, hypoglycemic drugs and strips should be given to people with diabetes. Macrolevel awareness programs should be initiated to ensure that people with diabetes know self‐care management, attend regular checkups and obtain medications from the hospital to keep their diabetes under control. Hospital physicians should provide adequate screening and health counseling for risk patients. The government should improve care delivery efficiently by reorganizing and strengthening the primary health care system. This enables people to access the health system through respective primary care, which helps easy tracking and tracing of patients' diabetic control. To increase the fairness and quality of the care given, cost‐effective protocols must be developed to make fair judgments during management. In the 2023 budget Government should increase the financial allocation for health care more than the previous year and reduce nonessential sectors' funding. The WHO's commitments toward diabetes are realistic targets and much needed in the context of Sri Lanka's diabetes burden. Government stability is the starting point for these goals in Sri Lanka.

## AUTHOR CONTRIBUTIONS

**Gajarishiyan Rasalingam**: Conceptualization; project administration; validation; writing – original draft; writing – review and editing.

## CONFLICT OF INTEREST

The authors declare no conflict of interest.

## TRANSPARENCY STATEMENT

The lead author Gajarishiyan Rasalingam affirms that this manuscript is an honest, accurate, and transparent account of the study being reported; that no important aspects of the study have been omitted; and that any discrepancies from the study as planned (and, if relevant, registered) have been explained.

## Data Availability

Not Applicable.

## References

[1] WHO . First‐ever global coverage targets for diabetes adopted at the 75th World Health Assembly. Published May 28, 2022. Accessed November 18, 2022. https://www.who.int/news-room/feature-stories/detail/first-ever-global-coverage-targets-for-diabetes-adopted-at-the-75-th-world-health-assembly

[2] Hannah E‐P , Devana S . People are going to die': crisis‐hit Sri Lanka runs out of medicine. The Guardian. Published May 31, 2022. Accessed November 19, 2022. https://www.theguardian.com/world/2022/may/31/people-are-going-to-die-crisis-hit-sri-lanka-runs-out-of-medicine

[3] Sarkar S . The devastating health consequences of Sri Lanka's economic collapse. BMJ. 2022;377:o1543. 10.1136/BMJ.O1543 35768079

[4] Matthias AT , Jayasinghe S . Worsening economic crisis in Sri Lanka: impacts on health. Lancet Glob Health. 2022;10(7):e959. 10.1016/S2214-109X(22)00234-0 35569487 PMC9098208

[5] International Trade Administration . Pharmaceuticals and Medical Equipment. Sri Lanka—Country Commercial Guide. Published September, 28 2021. Accessed May 25, 2022. https://www.trade.gov/country-commercial-guides/sri-lanka-pharmaceuticals-and-medical-equipment

[6] Thiagarajan K . Sri Lanka: doctors scramble for basic medicines as economic crisis engulfs nation. BMJ. 2022;377:o968. 10.1136/BMJ.O968

[7] World Bank . Sri Lanka Overview. Published October 6, 2022. Accessed November 18, 2022. https://www.worldbank.org/en/country/srilanka/overview

[8] Economy Next . Sri Lanka's price controlled drugs raised 14.4‐pct as central bank busts rupee. Published May, 2019. Accessed September 23, 2022. https://economynext.com/sri-lankas-price-controlled-drugs-raised-14-4-pct-as-central-bank-busts-rupee-14111/

